# Draft Genome Sequence of Vogesella oryzae L3B39^T^, Isolated from the Rhizosphere of Saline-Tolerant Pokkali Rice

**DOI:** 10.1128/MRA.00515-20

**Published:** 2021-02-04

**Authors:** Luis Johnson Kangale, Anthony Levasseur, Hussein Anani, Didier Raoult, Eric Ghigo, Pierre-Edouard Fournier

**Affiliations:** a Aix-Marseille University, IRD, AP-HM, SSA, VITROME, Marseille, France; b IHU-Méditerranée-Infection, Marseille, France; c Aix-Marseille University, IRD, AP-HM, MEPHI, Marseille, France; d Special Infectious Agents Unit, King Fahd Medical Research Center, King Abdulaziz University, Jeddah, Saudi Arabia; e Techno-Jouvence, Marseille, France; Broad Institute

## Abstract

Here, we report the draft genome sequence of Vogesella oryzae L3B39^T^ (CSUR Q2602^T^ = DSM 28780), which is a *Vogesella* species isolated from the rhizosphere of saline-tolerant pokkali rice. The genome sequence was assembled into 58 contigs for a total size of 3,415,129 bp, with a G+C content of 62.3%.

## ANNOUNCEMENT

**FIG 1:**
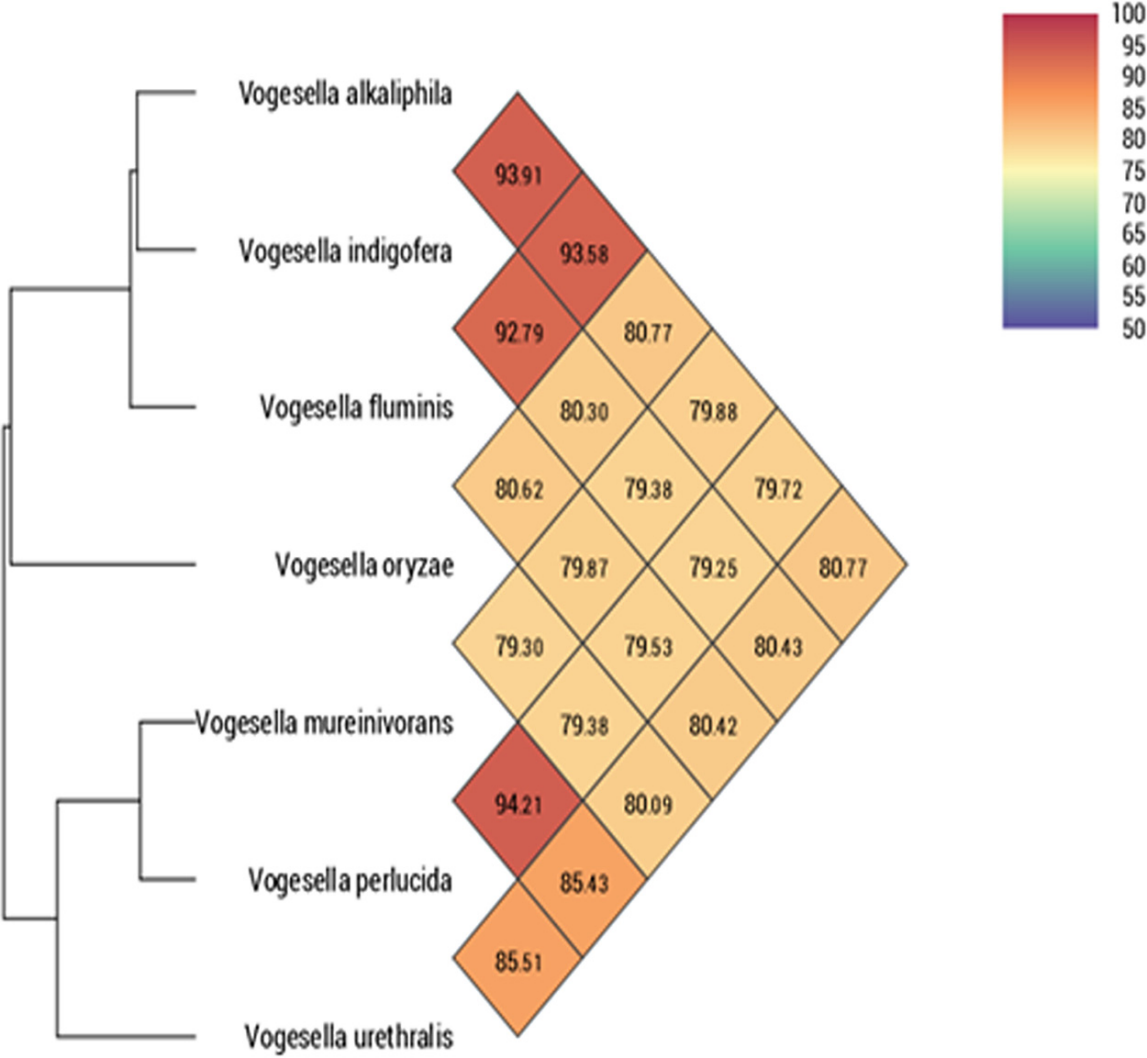
Comparison of strain L3B39^T^ with all available *Vogesella* species genomes. Values shown are percent identities.

The genus *Vogesella* was assigned to the family *Chromobacteriaceae* (1), with Vogesella indigofera as a type species (2). The strain L3B39 ^T^ has been isolated from the rhizosphere of saline-tolerant pokkali rice and is described as a type strain of Vogesella oryzae (3). Because the complete genomic sequence is not available, we have investigated the genomic features of this bacterium. Vogesella oryzae was grown for 24 h at 28°C on Columbia agar supplemented with 5% sheep blood (bioMérieux, Marcy l’Etoile, France). The genomic DNA (gDNA) of Vogesella oryzae L3B39^T^ was extracted via the use of an EZ1 automatic extractor and the use of a DNA tissue kit (Qiagen, Hilden, Germany). The gDNA was then quantified with a Qubit assay (Life Technologies, Carlsbad, CA, USA), and the concentration of bacterial gDNA was normalized to 0.2 ng/μl. The gDNA of Vogesella oryzae L3B39^T^ was sequenced using the mate-paired-end strategy with a MiSeq sequencer (Illumina, San Diego, CA, USA) (4). The gDNA was barcoded to be prepared with the Nextera XT DNA sample preparation kit from Illumina. First, dilution was performed to obtain 1 ng of gDNA as the input to prepare the paired-end library, and then “tagmentation” was performed. A limited-cycle PCR amplification (12 cycles) completed the tag adapters and introduced dual-index barcodes. The libraries were purified on AMPure XP beads (Beckman Coulter, Inc., Fullerton, CA, USA) and normalized on specific beads following the Nextera XT protocol from Illumina. Normalized libraries were pooled into a single library for sequencing on the MiSeq system. Automated cluster generation and paired-end sequencing (2 × 250 bp) with dual-index reads were performed in a single 39-hour run. Total information of 3.4 Gb was obtained from a cluster with a density of 354,000 clusters/mm^2^, with a cluster passing quality control filters of 96.5%. Within this run, the index representation for Vogesella oryzae L3B39^T^ (CSUR Q2602) was determined to 3.66%, and the sequence read length was 250 bp. The 6,897,812 paired-end reads of the MiSeq run were examined to evaluate quality using FastQC v0.11.8 (5). Sequencing reads for this strain were assembled using the SPAdes (6) genome assembler for regular and single-cell projects (Galaxy 3.12.0+galaxy1). The “careful” option was used to reduce the number of mismatches and short indels. Default parameters were used here and for all software (for k values, i.e., k-mer values of 127, 99, 77, 55, 33, and 21). SSPACE v1.0.7 (7) is a stand-alone scaffolder of preassembled contigs and was used to combine the contigs. The use of GapFiller v2.1.1 (8) allowed reliable closure of gaps within scaffolds. Manual finishing was performed based on similarity searches and synteny block detection using the sequence of Vogesella mureinivorans (9) strain 389^T^ (GenBank accession number NZ_VMTV00000000.2) as a template. The V. oryzae L3B39^T^ genome annotation was obtained using the NCBI Prokaryotic Genome Annotation Pipeline (PGAP) v4.11 (10). The genome of L3B39^T^ was assembled into 58 contigs (coverage, 5×; *N*_50_, 112,025 bp; *L*_50_, 12) totaling 3,415,129 bp, with a G+C content estimated at 62.3%. A total of 3,220 coding DNA sequences were predicted, along with 7 rRNAs and 69 tRNAs (Table 1). Using OrthoANI v0.93.1 (11), strain L3B39^T^ was compared to all available *Vogesella* species genomes. A significant difference was observed between genomes including strain L3B39^T^ (Fig. 1).

**TABLE 1:** Main genomic characteristics of Vogesella oryzae and other *Vogesella* species^*a*^

Species	RefSeq accession no.	Size (bp)	G+C content (%)	No. of contigs	No. of rRNAs	No. of tRNAs	No. of proteins	No. of genes
Vogesella mureinivorans	NZ_VMTV00000000.2	4,184,664	61.3	1,181	17	78	3,909	4,682
Vogesella indigofera	NZ_RBID00000000.1	3,621,567	64.3	20	10	69	3,333	3,454
Vogesella oryzae	NZ_CADEPL000000000.1	3,415,129	62.3	58	7	69	3,220	3,347
Vogesella perlucida	NZ_VOID00000000.2	4,327,579	59.5	903	11	68	4,028	4,204
Vogesella urethralis	NZ_VMDW00000000.2	4,028,666	63.2	157	4	59	3,670	3,774
Vogesella alkaliphila	NZ_BMYW00000000.1	3,418,988	65.1	29	6	72	3,190	3,271
Vogesella fluminis	NZ_BMYP01000000	3,300,252	63.5	124	5	62	3,021	3,147

a Data were taken from NCBI annotation.

### Data availability.

Vogesella oryzae strain L3B39^T^ is available at the CSUR under the reference CSUR Q2602 (= DSM 28780). The complete 16S rRNA gene sequence of Vogesella oryzae strain L3B39^T^ has been deposited in GenBank under the accession number KR363129. The genome sequence of Vogesella oryzae strain L3B39^T^ has been deposited in GenBank under the accession number CADEPL010000000. Illumina MiSeq paired-end sequencing raw data have been deposited under run accession number ERR4020027. The annotation is publicly accessible under GenBank accession number NZ_CADEPL000000000.
